# Identifying Challenges Related to the Management of Comorbidities in People with Dementia in Residential Care: Expert Delphi Consensus Exercise

**DOI:** 10.1177/07334648241309734

**Published:** 2025-01-06

**Authors:** Serena Sabatini, Frances Hawes, Kelechi Eluigwe, Eugene Y. H. Tang

**Affiliations:** 1 School of Psychology, Faculty of Health and Medical Sciences, 3660 University of Surrey, Guildford, UK; 2 Health Care Administration Department, 14747 University of Wisconsin Eau Claire, WI, USA; 3 Population Health Sciences Institute, 5994 Newcastle University, Newcastle upon Tyne, UK

**Keywords:** co-morbidity, multimorbidity, care home, nursing home, Alzheimer’s, health conditions

## Abstract

Improving early detection, management, and treatment of comorbid conditions to dementia in residential care could slow down cognitive and functional decline, and increase residents’ quality of life. We conducted a Delphi study comprising three rounds (two surveys and an interview) to identify the most difficult dementia comorbidities to deal with in residential care and related issues. Participants were 15 UK-based experts including academics, residential care workers, geriatricians, and neuropsychologists. In the first-round of the Delphi, experts mentioned 15 comorbid health conditions to dementia and 19 issues. In the following rounds of the Delphi mental illnesses, delirium, and sensory impairments were identified as the most difficult comorbidities to dementia to deal with. Medication management, symptom management, shortage of staff, lack of training among staff, and limited resources from the broader healthcare system were identified as the most difficult issues when dealing with dementia comorbidities.

**What this paper adds**

- Residential care staff, clinicians, and academics deemed mental illnesses, delirium, and sensory impairments as the most difficult dementia comorbidities to deal with in residential care.
- Residential care staff, clinicians, and academics deemed medication management, symptom management, shortage of staff, lack of training among staff, and limited resources from the broader healthcare system as the biggest issues when dealing with dementia comorbidities in residential care.

**Applications of study findings**

- To address disparities between different residential care homes there is the need of mandatory standards of care across UK residential care homes, and of clear guidelines on topics such as pathways of care for residents’ medical problems, staff to resident ratios, and minimum wages.
- Care assistants and nurses should receive better engaging, specific, and standardized training courses on mental illnesses, delirium, sensory impairments, and other key topics such as non-verbal communication.
- More investments in social care are needed to secure faster and continuous interaction between residents and external specialists, and to provide more specialized mental health institutions where more severe and aggressive residents can be moved.

## Introduction

Worldwide there are 55 million people with dementia with about 994,000 living in the UK, and this number is projected to increase significantly in the next decades (Alzheimer’s Disease International, 2023). Dementia refers to several neurological conditions involving significant decline in cognitive abilities, such as memory and language, that interferes with individuals’ ability to conduct everyday activities (American Psychiatric Association, 2013). Moreover, individuals with dementia typically experience one or more behavioral and psychological symptoms such as agitation and sleep disturbances (Vik-Mo et al., 2020).

About 90% of community-dwelling people with dementia have at least one additional comorbid condition; with the most common being hypertension, depression, diabetes, and inflammation affecting the joints (Dunn et al., 2022). Individuals with dementia with comorbid conditions receive less treatment and monitoring for non-dementia conditions (e.g., diabetes) than people with the same conditions but without dementia (Bunn et al., 2014). For instance, individuals with dementia undergo fewer health examinations, and hence have less likelihood of receiving new diagnoses (Pepper & Dening, 2024). Moreover, they receive fewer medications to manage existing conditions (Delgado et al., 2021). We hypothesize this may be due to dementia-specific challenges. Less examination among people with dementia may be because of their difficulty communicating with doctors. For example, they may be unable to provide essential feedback during an eye test. Fewer prescribed medications among people with dementia may be due to their high risk of experiencing side effects (Green et al., 2019).

Advancing evidence on the challenges related to early detection, management, and treatment of dementia comorbidities is essential to inform the healthcare system on the adjustments needed to address the complex needs of people with dementia (Livingston et al., 2020). This is a priority as the more comorbidities people with dementia have, the more they are at risk of faster cognitive and functional decline, poorer quality of life, and greater mortality (Bunn et al., 2014). Hence, better identification and management of dementia comorbidities would result in improved quality of life in people with dementia and their families, and in reduced healthcare cost. Research on the identification and management of dementia comorbidities in residential care is however scarce. This despite the fact that people with dementia living in residential care are those having more health issues. Indeed, while being in residential care, people with dementia often develop new health conditions and/or experience exacerbation of those conditions they already had when moving into care (Kovach et al., 2017; Ma et al., 2023).

When it comes to dealing with dementia comorbidities, residential care settings may bring extra challenges. Indeed, in residential care people with dementia are typically frailer, have poorer memory, and have more limited communication than those living in the community. These characteristics may make identification of new health conditions particularly difficult as residents may be unable to communicate health issues (e.g., pain) or to attend visits in hospitals. Moreover, in those agencies where care staff attend different residents at each shift, lack of familiarity with residents makes it difficult for them to detect behavioral changes and their underlying health issues. More research on residential care settings is essential as in the UK 39% of people with dementia live in residential care, and in the residential care population 70% have cognitive impairment (Alzheimer’s Society, 2014). The current study aims to seek the opinions of UK-based dementia experts (1) to identify what are the comorbid conditions to dementia most difficult to deal with in residential care and (2) what makes dealing with dementia comorbidities in residential care settings difficult. It is important to note that while this paper uses the term residential care as an overarching term, in the UK several types of residential care are available.

First, residential care homes provide accommodation, meals, personal care, and social activities. Second, nursing homes offer all the services of a residential care home, along with 24-h nursing care. Third, dementia care homes offer secure environments and staff trained on dementia-related behaviors. Fourth, assisted living combines independent living with access to on-site care and support services. Fifth, sheltered housing are self-contained flats/bungalows within a complex that offer shared facilities and an on-site warden to relatively independent seniors. Sixth, continuing care retirement communities offer several care options within one location, from independent living to full nursing care, allowing residents to transition to different levels of care as their needs evolve. Seventh, palliative care homes provide specialized care for individuals nearing the end of life, focusing on comfort and quality of life. In the initial stages of dementia individuals can reside in all these types of residential care settings, but when dementia progresses and their needs increase, they generally reside in a nursing or dementia care home^1^.

## Methods

### Research Design

We adopted a Delphi approach (David & Roberta, 2020) because of its iterative nature. Delphi studies are researches based on several rounds where a group of experts is asked their opinion on a matter. The questions experts are asked in each round are based on the findings of the previous round that are shared with experts prior to the next round. For this study, we decided a-priori that three rounds would have been necessary. In the first-round, occurring in the first two weeks of June 2024, participants were asked via email to (1) list those comorbid conditions to dementia that they find difficult to manage in residential care and (2) list issues related to dealing with dementia comorbidities. In the second-round, occurring in the last two weeks of June 2024, participants were asked to (1) read the list of comorbid conditions and pick the most difficult to manage. (2) read the list of identified issues and pick the most salient. This round allowed participants to reconsider their opinions based on those of the remaining participants. In the third-round, occurring in July 2024, those participants who gave availability were interviewed online using Microsoft Teams. Interviews were semi-structured and lasted between 20 and 60 minutes with an average length of 40 minutes. For those comorbid conditions identified as the most difficult to manage participants were asked to elaborate on the difficulties related to these conditions, and on what could be done to improve their management. Next, for the issues identified as the most common when dealing with dementia comorbidities in nursing homes, participants were asked to describe their causes and how they could be minimized. Supplemental text 1 reports the interview script.

## Participants’ Recruitment

Between 03/24-05/24 UK-based academics and practitioners were recruited via email. Inclusion criteria for academics were having ≥ five years of research experience and peer-reviewed publications on dementia comorbidities. Inclusion criteria for practitioners were having ≥ five years of work experience with people with dementia. Academics were identified through examination of their profiles and signposting via other academics. Expert practitioners were identified via emails to randomly identified residential care managers; the Kent, Surrey, Sussex Academic Health Science Network; and signposting by other practitioners. Thirty experts were invited to participate in the study, half academics and half practitioners. Fifteen participated to the first-round of the Delphi and eleven to the subsequent rounds. Four participants were solely working in academia as research fellows or professors; two were both professors and practicing experts (psychiatrist and neurologist, respectively); one was an old age psychiatrist; one was a geriatrician; one was an orthoptist; two were nurses; three were care assistants; and one was registered nursing home manager. This study obtained favorable ethical approval from the Ethics Committee of the University of “blinded” (Ref: “blinded”). No monetary reward was associated with study participation.

### Data Analysis

Interviews were video-recorded, manually transcribed, and analyzed using thematic content analysis (Allen et al., 1997). To ensure reliability, two researchers (SS; KE) independently completed the coding process. First, all transcripts were read and initial themes were identified. Second, the two researchers convened to discuss their observations and pinpoint shared themes and subthemes. The resulting list of themes and subthemes was used as a coding framework to code the whole dataset. Finally, they identified sample quotes to illustrate themes and subthemes. Analyses of interview data suggested data saturation as similar answers were provided by multiple participants.

## Results

### Comorbid Conditions to Dementia Most Difficult to Deal With

In the first-round of the Delphi participants reported 15 dementia comorbidities that they find difficult to manage in residential care (Table 1). In the second-round of the Delphi the comorbid conditions deemed as most salient were mental illnesses; delirium; and sensory impairments (chosen by eight, six, and four participants, respectively). These conditions represented the three main themes identified in the third-round of the Delphi (Table 2). Supplemental Table 1 reports sample quotes for the identified themes and subthemes.

**Table 1.** Delphi Study Round 1: Comorbid Health Conditions to Dementia.

Conditions mentioned
Mental illnesses in general and specifically anxiety, depression, bipolar disorder, schizophrenia
Diabetes
Stroke / Heart failure / Angina / transient ischemic attack (TIA) or stroke / Coronary heart disease (CHD)
Sensory impairment (hearing and visual)
Musculoskeletal conditions (i.e., joint disorders, osteoarthritis, and osteoporosis)
Chronic obstructive pulmonary disease (COPD), asthma, respiratory conditions
Kidney failure and electrolyte imbalance
Parkinson’s disease
Hypertension / high blood pressure
Infection particularly, urinary trait infection
Managing continence and stoma care
Delirium
Cancer
Skin disorders
Asthma
Other health issues mentioned
Pain, including tooth pain
Falls
Constipation
Eating and drinking and swallowing food (choking)
Frailty, weakness/impairment to mobility
Behavioral and psychological symptoms of dementia

**Table 2.** Themes and Subthemes for the First Study Aim.

Themes	Subthemes
Mental illnesses	Condition-specific challenges
	Issues that worsen detection, treatment, and management of mental illnesses
	What makes/could make management of mental illnesses easier
Delirium	Condition-specific challenges
	What makes/could make management of delirium easier
Sensory impairment	Condition-specific challenges
	Issues that worsen management of sensory impairment
	What makes/could make dealing with sensory impairments easier

#### Mental Illnesses

Mental illnesses comprised three subthemes: condition-specific challenges; issues that worsen detection, treatment, and management of mental illnesses; and what makes/could make management of mental illnesses easier.

The three main *condition-specific challenges* that were mentioned are as follows. First, among residents with dementia, depression is often undetected/underdiagnosed and consequently untreated. This can be due (i) to the similarity between depressive and behavioral symptoms of dementia; (ii) people with advanced dementia being unable to verbally communicate low mood; and (iii) withdrawn residents not being disruptive to care staff. Second, due to dementia-specific challenges and to limited social encounters in residential care, staff find it hard to meaningfully engage people with dementia. This often leads to preferring (sedative) medications over non-pharmacological interventions. Third, medications for depression in people with dementia may not only be ineffective but can also have serious/limiting side effects and bad interaction with other medications.

The following *issues that worsen detection, treatment, and management of mental illnesses* were mentioned. First, lack of specific knowledge and training among care staff (e.g., on the delivery of non-pharmacological interventions). Second, limited availability of mental health specialists and of general practitioners (GPs), which leads to long waiting times for medical consultations. Third, often the same resident is visited by a different specialist at each consultation, impairing continuity of care. Fourth, high staff turnover leads to hiring of inexperienced staff that are unable to detect unusual moods in residents as they are not familiar with them. Lack of familiarity with a specific resident also makes staff unable to know which activities, topics of discussion, and objects could lift the mood of residents.

Participants also mentioned *what makes/could make management of mental illnesses easier.* First, for new residents, to discern whether behavioral changes are due to the new environment or to long-standing mental illnesses it is essential to observe them a few days and consult their family and medical records. Second, external specialists coming into the residential care home to train the staff on non-pharmacological interventions are needed. Third, alongside more structured non-pharmacological interventions, activities that increase residents’ engagement (e.g., walking in the garden) would help addressing mental illnesses, and should be proposed before prescribing medications to residents. Fourth, care staff needs to better adhere to residents’ management plans as this could prevent their transition into a more specialized care setting or into a psychiatric ward. Fifth, there is the need of more specialist institutions where, when necessary, residents with dementia and severe mental illnesses can be admitted as, when aggressive, they can be dangerous for other residents. Sixth, when communication is preserved, asking residents about their mood may provide insight into their mental state. Seventh, taking the time to lift the mood of a resident (e.g., through small conversations) first could ease the performance of care-related tasks.

#### Delirium

Delirium comprised two subthemes: condition-specific challenges and what makes/could make management of delirium easier.

Four *condition-specific challenges* were reported by participants. First, because delirium can have many physical causes, identifying its cause can be challenging. Second, detection of hypoactive delirium, which is a subtype of delirium where people become withdrawn, is infrequent and can have dangerous consequences (e.g., dehydration). Third, even more common forms of delirium can be undetected as their symptoms are similar those of dementia. Again, this is dangerous as untreated delirium can reinforce dementia. Fourth, medications to treat delirium in people with dementia are of dubious efficacy.

Many strategies *make and/or could make management of delirium easier.* Some strategies are obvious, such as checking more frequently residents with delirium; communicating episodes of delirium to residents’ families; and having residents cared for by the same staff member as this helps when the resident is confused. As delirium is generally caused by a variety of medical issues (e.g., infections and dehydration), better prevention of these would decrease episodes of delirium. Moreover, when it comes to prescribing medications for delirium, medications’ side effects should be considered, and residents’ wellbeing should be prioritized. Participants also highlighted the need of changes in the healthcare systems such as the need of better and faster interaction between residential care staff and external specialists who prescribe medications. Participants also acknowledged the urgency to have specialists (e.g., clinical psychologists) training care staff on delirium. Moreover, clearer guidelines are needed as standardized pathways of care for residents’ medical problems would make clearer when residents should remain in residential care and when they should instead be hospitalized.

#### Sensory Impairment

Sensory impairment comprised three subthemes: condition-specific challenges; issues that worsen management of sensory impairment; and what makes/could make management of sensory impairments easier.

Participants mentioned two main *condition-specific challenges.* First, due to dementia-related limitations (e.g., language barriers) undertaking checks for sensory impairments is complex. Second, untangling sensory impairment from dementia-related understanding and communication difficulties is difficult.

Participants also mentioned several *issues that worsen management of sensory impairment.* First, even when staff members know that somebody has an impairment, it is easy to underestimate how disabling it is. Second, certain environments can amplify residents’ sensory impairments (e.g., noisy environments). Third, although participants reported that both GPs and specialists for visual impairment go into nursing homes to examine residents, the waiting time is very long. Fourth, the hearing aid market is not tailored to people with dementia. Fifth, staff compliance with care plans is low. Indeed, often care staff do not know how to use hearing aids, how to change their batteries, or lose them.

At the same time participants suggested *what makes/could make dealing with sensory impairments easier.* First, to ameliorate detection of sensory impairments, easier and faster interaction between residential care homes and specialists (e.g., speech therapists) is needed. Second, to improve management of sensory impairments, continuity of care would allow care staff to know residents and their preferred way of interacting. Third, it is important to personalize interactions with residents with sensory impairment (e.g., shout more) and elements of their environment/room (e.g., having big signs). Fourth, simply reminding residents to wear hearing aids can make a significant change as they may forget to wear them, be scared of them, or lack awareness of their impairment. Fourth, there is the need of specific training for care staff on non-verbal communication. Finally, it is important to give staff permission to trial several strategies until they find the best way to communicate with the resident.

### Most Salient Issues when Dealing with Comorbid Conditions to Dementia

In the first-round of the Delphi participants reported 19 issues related to dementia comorbidities (Table 3). The most salient issues identified in the second-round of the Delphi were symptoms management; medication management; shortage of staff; and limited skills among residential care staff (each chosen by five participants). In the third-round of the Delphi five themes were identified: medication management, symptoms management, shortage of staff, limited skills among staff, and limited resources from the broader healthcare system (Table 4). Supplemental Table 2 reports sample quotes for the themes and subthemes.

**Table 3.** Delphi Study Round 1: Issues When Dealing With Dementia Comorbidities.

Issues mentioned
Communication issues in the person with dementia which undermines communication of symptoms and the ability to choose for medical treatments
Management of symptoms (e.g., breathlessness, pain, and falls), behaviors (e.g., aggression and agitation)
Care homes are isolated within the wider network of services. Lack of point-of-care tests available (i.e., test that could be taken into the care home). People with dementia may miss out on regular eye tests because they are not put forward for them. Lack of dedicated secondary care services going into care home. Still, these residents have higher need of ambulatory and inpatient care
Limited skills among staff to recognize and address comorbidities (e.g., patients with dementia need a lot of supervised walking to retain their mobility, a lot of 1-to-1 attention to address episodes of low mood or anxiety). Residential homes do not have qualified nurses on shift so are more reliant on external professionals. Lack of understanding of the complexities of treating someone living with dementia
Patients are rarely taken to the hospital for checks/test for several reasons and this leads to lack of diagnosis of new comorbidities. Attending appointments at hospital or specialist clinics which are not ideal for those living with dementia or getting professionals to attend the sufficient and/or skilled staff to provide adequate support. Access to healthcare professionals to support management of long-term conditions (LTCs/comorbidities)—GPs, district nurses, practice nurses, and other community support may not be accessible. Ability to access diagnosis and secondary care for medical conditions, for example, can be too challenging to attend outpatient appointments and attend hospital for formal investigations and to see specialists and so CH residents may miss out on specialist care. On the other hand, conditions may be easier to manage if CH staff are able to access external specialist practitioners who come into the home, for example, diabetes nurses
Medication management including finding appropriate medication; understanding whether that medication is suitable or will increase some symptoms of dementia or cause more harm than good/decrease quality of life; timing of medications; deprescribing; interactions between medications for different conditions; and side effects
Limited staff and time among staff
Functional status
Increased burden on staff
Staff knowledge of the conditions and how to manage these, for example, this may not be communicated adequately in information on admission to CH, or for conditions diagnosed after and via care plan
Risks management, for example, pressure sores and falls
Awareness issues in the person with dementia which undermines compliance with treatment
Nutritional and hydration challenges: Comorbidities such as diabetes, heart disease, or swallowing disorders can complicate the nutritional and hydration needs of dementia patients
Managing families’ expectations about outcomes for this person
The unfamiliar environment can exacerbate symptoms
Type of dementia is not clear in many of the cases in care home
Some health issues (i.e., infections) can exacerbate with and interact with the person’s mental function
Provision of emotional support
Decline in residents’ quality of life

**Table 4.** Themes and Subthemes for the Second Study Aim.

Themes	Subthemes
Medication management	Discrepancies between residential care settings
	Challenges
Symptoms management	
Shortage of staff	Causes of staff turnover
	Discrepancies between residential care settings
Limited skills among staff	Causes of limited skills among residential care staff
	Strategies to upskill residential care staff
Limited resources from the broader healthcare system	

#### Medication Management

Medication management comprises two subthemes: discrepancies between residential care homes and challenges. First, we noticed *discrepancies between residential care homes*. Indeed, participants’ answers suggest different frequencies with which residential care homes provide review of medications/drugs.^2^ Some experts stated this is done every six months, others every year, and others that this is never done. Moreover, whereas some residential care homes provide a medication for every health issue the resident has, others upskill their staff so they can avoid medications as much as possible and instead deliver non-pharmacological interventions.

Participants also elaborated on the *challenges* related to medication management. The most common challenge is limitation of medications side effects, which are frequent in people with dementia. A challenge related to this is deprescribing^3^, especially at care home admission, as many medications as possible. Indeed, often residents are on preventive medication (e.g., statins) that are of no value to them given their life expectancy. Reviewing residents’ medications would be an opportunity for deprescribing medications that are no longer needed or that lead to severe side effects. However, period medication reviews do not occur everywhere. Although pharmacists would be more specialized for this task, participants said this task is frequently undertaken by GPs. Finally, convincing residents to take medications, even in cover form, may not be easy.

#### Symptoms Management

Symptoms management does not have subthemes. Managing symptoms in people with advanced dementia is difficult as they are unable to communicate pain to staff. Behavioral and psychological symptoms of dementia are often used as a pain indicator. To investigate whether behavioral issues are due to pain in some residential care homes, people with dementia are given paracetamol and, if the behavioral symptom disappears, it is inferred that they are in pain. Non-pharmacological interventions are also useful to alleviate pain, but these are rarely provided to residents with dementia due to the (erroneous) assumption they would not work with them. Although participants reported that GPs can advise on symptoms management, the waiting time takes up to two months. Finally, among residents having several symptoms, care/medical attention should be focused on those symptoms that impact the most residents’ quality of life.

#### Shortage of Staff

Shortage of staff comprises two subthemes: causes of staff turnover and discrepancies between residential care homes. Participants mentioned several *causes of staff turnover* including high job-related burden; low pay; taking the care assistant role as a momentary job experience; and lack of “the right attitude” to work with people with dementia. Despite the already high turnover among UK care staff, one participant mentioned that staff turnover would be even higher if some care workers would not be sponsored by the agencies. When asking participants to comment about shortage of staff in residential care, we found discrepancies in their views, suggesting *discrepancies between residential care homes.* Whereas some agencies choose the number of staff members based on a given residents-staff member ratio; others choose the number of staff members based on residents’ needs at different times of the day in a way that during more demanding hours more staff are present. However, whereas some participants complained about shortage of staff where they worked others did not.

#### Limited Skills

Limited skills among residential care staff comprise two subthemes: causes for limited skills among residential care staff and strategies to upskill staff members. Participants reported several *causes for limited skills among residential care staff.* First, turnover undermines retention of experienced and knowledgeable staff. Second, limited staff impacts on the delivery of in-person training courses because staff members have to attend residents rather than courses. Third, training courses often focus on priorities like insurance requirements rather than on care-related matters. Moreover, to refresh staff knowledge, often staff undertake the same courses every year. This means training courses rarely go beyond basic concepts. Fourth, training courses vary across agencies: whereas few agencies provide advanced training, others provide only basic training. Fifth, staff may not pay attention to training courses, due to these failing to engage attendees. Sixth, the limited budget for social care (e.g., to pay external trainers) impacts on the quality of courses delivered. Finally, often knowledge is passed down from more senior to junior staff. This can be an effective ecological strategy when the senior staff are knowledgeable, but it can backfire when senior staff have been perpetuating wrong behaviors.

Participants also mentioned *strategies to upskill residential care staff* including provision of a three-month induction, followed by continuous training. Courses should be enjoyable, fun, and delivered by specialists. Role modeling done by support workers is also an excellent strategy to teach practical tasks.

#### Limited Resources from the Broader Healthcare System

Limited resources from the broader healthcare system has no subthemes. This theme highlights that what residential care homes can do to deal with dementia comorbidities is limited if the following changes do not happen in the broader healthcare system. First, there is the need of clear standards of care across UK residential care homes and of evaluators checking that these standards are consistently met. Second, better and faster interaction between residential care homes and the external healthcare system is required. Indeed, the waiting time of one to two months to receive a visit from GPs and other specialists is extremely long for frail older residents. Interaction between residential settings and the external healthcare system should also improve through provision of continuity of care, that is the same resident should receive subsequent visits from the same specialist. Third, there is the need of clearer guidelines on care pathways for medical problems that residents have in a way that it is easier for the residential care home and residents’ families to decide whether it is in the best interest of the resident to be hospitalized or to stay in residential care. Fourth, more specialist institutions where residents with dementia and complex mental illnesses can be translated are needed. Overall, more funding for social care are necessary.

## Discussion

This study sought the opinions of 15 experts to identify which comorbid conditions to dementia are most difficult to manage in residential care, and what are the issues related to their management. Starting from the first study aim, mental illnesses, delirium, and sensory impairments were identified as the most difficult comorbidities to deal with. Analysis of participants’ interviews highlighted several issues common across these three conditions. First, because their symptoms can be confused with those of dementia they are hard to diagnose (Dunn et al., 2022; Ma et al., 2023). Moreover, impaired communication in people with dementia further obstacles medical examinations and the pursuit of diagnoses. Involvement of GPs, community geriatricians, and other specialists are essential to timely detect these conditions in people with dementia. However, access to these professionals generally requires a long waiting time (Davies et al., 2011).

Although the above conditions and issues are also experienced by community-dwelling people with dementia (Zazzara et al., 2022), some additional issues are specific to residential care settings. First, even though UK GPs are commissioned to look after residential agencies on their patch, continuity of care is infrequent. Moreover, despite mental illnesses, delirium, and sensory impairments being frequent in people with dementia, residential care staff know little about these conditions (Smythe et al., 2017). In addition, not allowing care staff to consistently assist the same residents and to personalize their approach of care to the resident’s needs obstacles identification and management of these conditions (Ray et al., 2019). Finally, some care staff members were skeptical about the ability to deliver structured non-pharmacological interventions to cognitively impaired individuals. This confirms that although effective non-pharmacological interventions for individuals with dementia have been developed, they are rarely used to treat depression and delirium in residential care settings (Meyer & O’Keefe, 2020). Hence, there is the need of greater transfer of knowledge between academics and residential care homes.

Moving to the second study aim, the most difficult issues when dealing with dementia comorbidities in residential care were medication management, symptom management, shortage of staff, lack of training among staff, and limited resources from the broader healthcare system. First, regarding medication management, discrepancies between agencies emerged in the frequency with which residents’ medications are reviewed. This may be due to the vague UK guidelines that recommend medications reviews to occur at least once a year. Aligned with previous evidence, residential care homes also differed with regard to the facility with whom they provide medications to residents (Ibrahim et al., 2021). Second, management of behavioral symptoms was deemed challenging due to having to identify their underlying medical issues in residents with limited communication (Atee et al., 2021) and having to wait a long time before receiving advice from specialists (McIntyre & Chow, 2020).

Third, another issue when dealing with dementia comorbidities is shortage of care staff which is due to high staff turnover (Commission, 2012). Unsurprisingly, high job-related burden, low pay (Bimpong et al., 2020), undertaking the care assistant role as a momentary job, and lack of the right attitude to work with people with dementia were mentioned as reasons behind high staff turnover. Courses aimed to decrease job-related burden in care staff have been developed and should be made available more broadly (Smythe et al., 2017). Imposing a minimum wage for care assistants could be a first step towards addressing discontent among care staff due to low pay and the significant disparity in the pay of care staff between UK agencies. To combat shortage of staff, sponsorship provided by agencies to care staff could make the job more attractive to non-UK citizens. However, this could backfire as care staff may avoid resigning even when they are unsatisfied with their job and their emotional state can negatively influence the quality of care provided. Finally, hiring staff who aims for a career as a carer and with the right attitude seems a tricky task for residential care homes as those who apply for care assistant positions are mostly unexperienced young individuals who know little about older people and dementia. Hence, once hired, psychoeducation courses should be delivered to sensitize staff to older age and dementia. Also with regard to shortage of staff in residential care homes, we found discrepancies in participants’ views. Whereas some wished there were more staff members per resident, others were happy with the number of staff in the agency where they worked. The Care Quality Commission, the regulatory body that governs UK Residential Homes, does not state any specific “care staff per resident requirement.” Hence, the care staff per resident decision lies entirely with the home’s manager. Whereas many agencies use a spreadsheet to keep track of their care staff per resident requirements, tailored to their specific needs; others have a fixed staff per resident ratio.

Fourth, limited skills among staff was another issue related to management of dementia comorbidities. This is due to high staff turnover; limited staff; lack of advanced, engaging, and standardized training courses across residential care homes; and bad modelling. In contrast, provision of a three-month induction to new staff; advanced and enjoyable training delivered by experts; and exposition to good role modeling are optimal strategies to upskill staff. Upskilling residential care staff on geriatric and clinical knowledge emerged as essential in this study as some of the identified issues such as medication and symptoms management would be more easily manageable if care staff would be adequately trained (Como et al., 2024). Previously proposed approaches to upskill care staff are requiring nurses to submit portfolios each year evidencing education in their fields to the Nursing and Midwifery Council and requiring residential care nurses to have experience in geriatric populations while training before graduating (Banerjee et al., 2017). Future research could also focus on co-production of training courses to make them attractive to residential care staff.

Fifth, what residential care homes can do to deal with dementia comorbidities is limited if a change does not happen in the broader healthcare system. To address disparities across agencies there is the need of standards of care across UK residential care homes, and of clear guidelines on topics such as pathways of care for residents’ medical problems, the staff to resident ratio, a minimum wage, and training requirements in residential care staff. Despite this, staff should be encouraged to personalise their approach of care based on residents’ needs. Moreover, more funding should be put into social care to increase the number of healthcare workers and, consequently, make interactions between residential settings and external specialists and institutions faster, effortless, and delivered in a way that ensures continuity of care (Sandvik et al., 2022).

A limitation of this study is its focus on the UK context, limiting generalizability of results to other countries. Cross-cultural studies are needed to understand which issues are common across countries. Second, this study focused on all residential care settings. Future research could investigate whether issues related to management of dementia comorbidities vary across different residential care settings. Third, although 15 experts participated in the first-round of the Delphi, only eleven participated to the optional interviews. Fourth, the study is based on a convenience sample and we cannot exclude selection bias.

## Conclusion

According to 15 dementia experts, mental illnesses, delirium, and sensory impairments are the most difficult dementia comorbidities to deal with in residential care. Moreover, the most difficult issues when dealing with dementia comorbidities in residential care were deemed medication management, symptom management, shortage of staff, lack of training among staff, and limited resources from the broader healthcare system. We need to reflect on these issues to develop strategies to improve detection and management of comorbidities in residents with dementia, leading to reduced stress in staff and increased quality of life in residents with dementia and their families.

## Supplemental Material

Supplemental Material - Identifying Challenges Related to the Management of Comorbidities in People with Dementia in Residential Care: Expert Delphi Consensus Exercise Supplemental Material for Identifying Challenges Related to the Management of Comorbidities in People with Dementia in Residential Care: Expert Delphi Consensus Exercise by Serena Sabatini, Frances Hawes, Kelechi Eluigwe, and Eugene Y. H. Tang in Journal of Applied Gerontology

## Notes

1 In the UK, individuals’ permanence in residential care can be funded by a local authority, self-funded, or (partially or fully) funded by the National Healthcare System (NHS). Those individuals who do not own a house and with savaging inferior to £23,250 are eligible for NHS fully funded residential care. However, individuals with a significant physical condition (in addition to dementia) get a portion of their residential care fee covered by the NHS (e.g., £235.88 a week for England).

2 Medication reviews consist of a structured, critical examination of a person’s medicines by a healthcare professional. It includes the person’s views and understanding about their medicines. Where appropriate, **(**e.g.**, when the patient/resident has little to no capacity)** it also includes family members or carers. The healthcare professional answers concerns, questions or problems with the medicines, including side effects or reactions. Finally, it should happen at least once a year but more often based on the person’s health and care needs (https://www.cqc.org.uk/guidance-providers/adult-social-care/primary-care-medicines-support-people-living-care-homes; https://www.nice.org.uk/guidance/sc1/resources/managing-medicines-in-care-homes-pdf-61677133765).

3 De-prescribing is a process of safe and effective reduction or stopping of inappropriate medicines. The aim is to reduce harm and the use of unnecessary medicines. **Reductions of medications is generally discussed with the patient/resident and/or their family members (based on the capacity of the patient/resident)** https://www.cqc.org.uk/guidance-providers/adult-social-care/primary-care-medicines-support-people-living-care-homes.
