# Supplementary material for: Identifying Challenges Related to the Management of Comorbidities in People with Dementia in Residential Care: Expert Delphi Consensus Exercise
Source: J Appl Gerontol. 2025 Jan 6;44(9):1503–12. doi: 10.1177/07334648241309734 (PMC12335622; doi:10.1177/07334648241309734)
Supplement: Supplemental Material - Identifying Challenges Related to the Management of Comorbidities in People with Dementia in Residential Care: Expert Delphi Consensus Exercise [file sj-pdf-1-jag-10.1177_07334648241309734.pdf]

Supplementary Table 1. Quotes Supporting Themes and Subthemes Related to Comorbid Conditions

THEME: MENTAL ILLNESSES

Subtheme: Condition-specific challenges

I think a lot of people get misdiagnosed with depression in the first instance when they're starting with symptoms of dementia (Research fellow).

It's also difficult to tease it [depression] apart from apathy, so obviously most people in care homes have to have dementia, whether it's diagnosed or not (Academic old age psychiatrist).

I think that talking therapies are really difficult for people with dementia, like the things that you would do for somebody that hasn't got dementia, like cognitive behavioural therapy, things like that they just probably [are] not going to work (Research fellow).

Older people in care homes with dementia and other comorbidities may have problems with antidepressant side effects (Academic old age psychiatrist).

People come [at nursing home admission] on five different psychiatric medications to us and then the majority of them is [sic] shown to have in research settings no benefit (Old age psychiatrist).

Subtheme: Issues that worsen detection, treatment, and management of mental illnesses

The main point for mental health issues is of course just [residential care] staff training and staff awareness (Old age psychiatrist).

When you do a referral [refer a patient to a specialist] to get a response or to get any support from the GPs [it] is so long winded (Senior care assistant).

Once you do get support from a GP, the next time you need support, it's a different person [different GP] (Senior care assistant).

All levels of permanent staffing are on such high staff turnover that there's no kind of continuity and the kind of staff don't get to know the residents, which I think is often crucial in dealing with most of these problems [comorbidities] really (Academic old age psychiatrist).

Try more psychosocial strategies and then eventually move to medications and try to find the fine balance (Registered nurse).

Reminding a person with dementia that they have dementia all the time, or them [care assistants] reminding them [residents with dementia] of a previous trauma all the time and that will often escalate the situation. It will lead to agitation or even depression (Old age psychiatrist).

Well, I think if you have the right team… some people [care assistants] don't like working with dementia and find it really difficult (Senior care assistant).

Subtheme: What makes/could make management of mental illnesses easier

The main thing that you have to do in this population [residents with dementia] is kind of really the non-pharmacological interventions, how you approach those people, how you deal with them (Old age psychiatrist).

I guess moving people [with dementia] around between institutions is sometimes necessary. If

people have got behaviour problems, then they may end up in a more specialised facility or somewhere with slightly better staffing (Academic old age psychiatrist).

There is not enough institutions in England to provide direct support to support people with mental health problems. Therefore, the nursing home, we have lots of residents with those conditions and clearly sometimes that impose a danger to other residents as well. When somebody has an aggressive type of behavior, they should be in more specialistic placement (Registered manager).

They [mental health specialists] would develop behavioural management plan. But then it depends very much on the staff and the care home to take up that plan at all (Old age psychiatrist).

Then we're starting a process of reassessing and working with that particular resident or family member to understand if this is the normal or this is something new and we work in collaboration with community mental health team. If we don't see medical summary, we would not often be told that somebody might be having a bipolar [disorder] as well or other, you know, mental health disorders (Registered manager).

To find the correct medication that suits their [people with dementia] needs best. Just the regular receipt on a puzzle, it doesn't quite crack it with residents that are anxious, depressed, frightened. It doesn't help with that at all that situation. But I do find that stimulation plays a big part activity trying to get them engaged and stuff, even if it's just a walk in the garden, it can have a real stimulus to them and they do calm down from that (Senior care assistant).

Ask people [residents with dementia] about their mood or to say you know you look a bit sad or you look a bit down today (Academic old age psychiatrist).

So it's not all about not having the time. The other thing is that having the time, well, if somebody's depressed, then it may take longer to complete care tasks with them. So actually, if you put the time in earlier to help them not be depressed and actually you'll be able to complete your task quicker. So in fact you might save time overall by investing a bit earlier on (Academic old age psychiatrist).

The initial thing is help to implement non pharmacologic interventions. So that's the idea. And then if the care home engages with that and is involved with that, that would be the kind of ideal scenario because that would probably solve their [residents with dementia] problem. But if they don't do that I guess the next way might then be getting the psychiatrist involved and prescribing sedative medications (Old age psychiatrist).

THEME: DELIRIUM

Subtheme: Condition-specific challenges

A lot of the delirium we saw was brought about by things like a urinary trait infection (Research fellow).

In older people it can just be sometimes difficult to identify what has triggered it [delirium] because it can be sometimes very little. It can just be, you know, they haven't been to the toilet for three days or a change in the environment. And I guess sometimes we just don't know what brings it on (Old age psychiatrist).

Quite a lot of people have hypoactive delirium where they just become withdrawn, and they're

easy to miss [in residential care]. I mean, they're often missed in hospital as well (Academic old age psychiatrist).

I would say that delirium is easy to confuse again with dementia (Research fellow).

I think that a lot of people present with sort of anxiety and I also care for people that had sort of more severe mental ill health like bipolar and schizophrenia as well, which again, that just makes the dementia, worse. And then it sort of goes in a cycle (Research fellow).

We sometimes give sedative medications to make them [residents with dementia] appear less distressing, but we actually don't know if they're really less distressed (Old age psychiatrist).

Subtheme: What makes/could make management of delirium easier

I would say maybe when somebody does have delirium and it is to such an extent that you need to monitor them, probably give them extra fluids. It's just the extra measure that you need to put in place to check more regular on the person and to provide more support (Registered nurse).

I think there should be an additional training looking into mental health (Registered manager).

I think clinical psychologists certainly in the old days with the psychologists were quite good at training people [residential care staff] in behavioural management. But of course, there may not be enough of those people [clinical psychologists] to input into all care, so you need care experts who have been trained in those sorts of aspects, so identifying antecedents, behaviour, consequences (Emeritus professor of psychology, and consultant clinical neuropsychologists).

They [residents with dementia] like that one specific person [care assistant]. They can be horrible to everyone around them, but they're lovely to that one person (Senior care assistant).

The first thing you do is either interacting with the family to understand whether there is indeed a change, and then you ask for external doctors and experts to come in (Registered manager).

Avoid resorting to ways of managing it [delirium] which will make it a very bad experience for the person (Emeritus professor of psychology, and consultant clinical neuropsychologists).

There's the interaction with the family and the family understanding what's going on to their loved one in terms of their behaviour (Emeritus professor of psychology, and consultant clinical neuropsychologists).

Nations should set out standards and those standards should be standards of care and they should be way of evaluating [residential care homes] (Emeritus professor of psychology, and consultant clinical neuropsychologists).

Clearer pathways of care for medical problems that people have so knowing when to admit them [residents with dementia] to hospital and when to it would be better to keep them in the care home (Geriatrician).

Making it easy for care homes to access advice from a GP.

It is very important to kind of avoid all minor triggers [of delirium]. So it is just very important that care homes keep an eye on adequate nutrition that people [residents] eat enough, drink enough, go to the toilet enough, have enough exercise, have all of those things (Old age psychiatrist).

THEME: SENSORY IMPAIRMENT

Subtheme: Condition-specific challenges

How does somebody [specialists] prescribe when someone [residents with dementia] can't communicate what they're seeing? (Research fellow).

Understanding and expressing language, and especially in the earlier stages, difficulty understanding language is often attributed to, like a hearing problem (Old age psychiatrist).

Subtheme: Issues that worsen management of sensory impairment

Even if people [care staff] know that somebody has got an impairment. It's easy to underestimate how disabling it is (Academic old age psychiatrist).

For sensory impairments, for sight, the lights in their homes are rubbish. They're too glaring which doesn't help somebody with a sensory impairment (Senior care assistant).

That's [hearing issues] made worse in noisy environments, which the common areas are quite noisy, so that makes things worse for people. Maybe television is blaring away and so forth (Academic old age psychiatrist).

The hearing aid market is not geared towards them [people with dementia]. I think because, like I said, they probably not [sic] need a fancy hearing aid (Old age psychiatrist).

Often there are problems with those [hearing aids], like the staff don't know how to use them, they don't know how to change the batteries, the hearing aids get lost (Academic old age psychiatrist).

That's about training of staff and monitoring staff compliance with care plans (Geriatrician).

I think [in the nursing home] we had a really good service. Specservice used to come (Research fellow who worked as a nurse).

Subtheme: What makes/could make dealing with sensory impairments easier

Getting to know the individual (Academic old age psychiatrist).

I remember having to scream in people's ears to get them to hear what we [care staff] were going to do, you know, it sounds awful, but you'd be sort of shy. I'm going to give you a wash right now and they then scream back. OK, you know, because they've just heard what you're going to do, but otherwise it could get that people would immediately lash out because you don't know what's happening (Research fellow).

We agreed to try something for a person [resident] at one point, you know, they couldn't see very well so we put some tape around the door handle and we made the frame around the door as well to make it more visible for this person to try and find a room (Registered nurse).

Making sure that people [residents] use equipment that they've been given either glasses or hearing aids. But once again when you have dementia you think what is this in my ear I don't want to have that (Registered manager).

I think that's about making sure people [residents] have their glasses and hearing aids put on, and you often hear stories of people in care homes and their hearing aids haven't been put in (Geriatrician).

If [residential care] staff felt that they have permission to do what they needed to do, then actually they could communicate quite effectively in whatever way that meant you know. So

whether that was about one to one or quiet time or using hearing aids at particular times (Academic old age psychiatrist).

I did some training sessions where we [care staff] wore glasses that were sort of damaged so that we could pretend that we had that visual impairment to give us a bit more sort of empathy [towards residents with hearing impairment]. You’ve no idea what it’s like to live like that. If that was one of the best training sessions I went on because I was like, Oh my goodness, that must be awful because you can't see anything straight ahead (Research fellow).

Lack of training is the major barrier. I have been within the care home setting with dementia for five years and I have to say that I had incredible training with the company that I first started with but not all companies are very good with their training for dementia. The company I'm with now is an example of that. Their training is not up to par, it's not engaging, it's not informative enough (Senior care assistant).

The nonverbal communication is probably just one of those major training areas for people [staff] in care homes (Old age psychiatrist).

So what would be good for residents with hearing issues is to get from our speech and language therapist a whole communication assessment but that's of course not routinely done in care homes in general because I guess often I mean there are questions of course for people where English is not their first language and then they develop dementia and then they they've stopped speaking maybe English but then the question is how much can they speak in the original language (Old age psychiatrist).

Supplementary Table 2. Quotes Supporting Themes and Subthemes Related to Issues in Dealing with Comorbid Conditions

THEME: MEDICATION MANAGEMENT

Subtheme: Discrepancies between residential care settings

Officially, we're [residential care settings] supposed to have a medication review with all of the residents every six months. So hopefully that then gives enough time to change anything if needed. But then there's been six months of a chance like as you say, that the medication even either hasn't been working or has been causing worse [side] effects (Senior care assistant).

They [residents] should technically of course have annual health cheques from the GPs, but I don't think that's necessarily defined that a medication review would be part of this and that just depends on how many community pharmacists there are (Old age psychiatrist).

Well, I think the management of medications is pretty good now because the surgeries have to follow the NICE guidelines…The care quality commission checks if every resident have had a medication review every six months and had blood tests and everything else. So I think it is much better than what it has been definitely (Senior Care assistant).

So medication normally is reviewed once a year by the doctor and in terms of having cover medications, we do review every month to see if there's any changes. Otherwise, every six months to one year because, you will need to redo the capacity assessment to see if anything has changed within the cognition of the resident (Registered nurse).

But ideally you would want to kind of upskill the care home workforce to deal with mental health problems better (Old age psychiatrist).

Subtheme: Challenges

Obviously, management of medication, even though it's uncovered, the resident might still choose not to have the glass of juice, might choose not to have the spoon of porridge where you're trying to cover the medication. So, then the symptoms may still come. They have high blood pressure; they need blood thinner medication because there are risk of stroke. So these problems may still persist, although you have everything in place. If the resident will still not take the medication in the cover form, you have to monitor for such kind of symptoms that can come from the lack of medication (Registered nurse).

The older people get the more sensitive they are to medication side effects.

To experience drug interactions (Academic old age psychiatrist).

To review or reduce and stop medications that are no longer needed. But there is the need to have a process in place to do this (Old age psychiatrist).

There are community pharmacy teams who sometimes go into care homes. That is … very patchy (Old age psychiatrist).

So, I mean, pharmacists are very good at doing this [medication reviews]. So there are some homes where pharmacists go and do, you know, sort of review everybody's medications. So they're pretty good (Academic old age psychiatrist).

THEME: SYMPTOMS MANAGEMENT

I think management of symptoms that detract from quality of life is the most important thing to stop in somebody from having pain, having sort of adverse symptoms like constipation or diarrhoea (Research fellow).

The only thing I would say is that sometimes a resident with severe dementia is unable to tell you if they're in pain or not. So if you find that they're distressed, it's a good idea to give them paracetamol regularly just to see if there's any difference. So then you know that if they calm down they were actually in pain because they would be unable to tell you (Senior care assistant).

Taking the dog around to see people [residents], like somebody brought a dog in so we take the dog around to see people and our children coming in and, doing jigsaws or knitting, it doesn't matter if it goes wrong (Research fellow).

They [GPs] do not have the capability to attend very fast, or sometimes it may take few weeks, a month, two months until they respond. Obviously when somebody has a severe mental health crisis, then we [nurses] pressure the doctor as well to follow up with them and we follow up with phone calls and then they [doctors] realise that this is urgent, so they start prioritising (Registered nurse).

THEME: SHORTAGE OF STAFF

Subtheme: Causes of staff turnover

We know that it's long hours, it's though work in a way it's not very well recognised by society (Old age psychiatrist).

I think it's the pay. Pay isn't high enough for the job we do (Senior care assistant).

Yes, I can tell you that at least 90% are not happy in terms of payment level (Health care assistant - dementia department).

I think you have to be a certain type of person to work within the care setting (Senior care assistant).

What I like about the living world with dementia is its positive approach to people with dementia, creating a positive attitude or vibe around it, and that hopefully will encourage good people to get into dementia care, including nursing, nursing staff and so forth, and realise it's the benefits, the positive aspects (Emeritus professor of psychology, and consultant clinical neuropsychologists).

It seems to be a really good first job [care assistant role] for young people, which isn't always what a care home needs as a setting (Senior care assistant).

If it wasn’t for sponsorship [visa sponsorship], I can tell you that there would be a mass resignation (Health care assistant - dementia department).

Subtheme: Discrepancies between residential care settings

I can't say that about where I work because we have a good ratio [referring to the ratio between staff and residents] (Senior care assistant).

So there's a lot of difference in the pay depending on which care home you're working on (Health care assistant - dementia department).

In the care home setting you have to provide care to so many people even though you know when you're looking into the ratio of the staff, preferably 1:1 would be ideal because then you could do that meaningful support and really support any type of behaviour but we know that's not the case

(Registered manager).

Based on the needs of our residents, we review them [residents' needs] every month and we see how many staff members we need during the day. In the morning, in the evening, at night, where we need more staff members or we do not need more so (Registered nurse).

THEME: LIMITED SKILLS AMONG STAFF

Subtheme: Causes of limited skills among residential care staff

Yes. Yeah, I think that one of the things that happens is sometimes bad habits get passed on as well. So sort of lifting techniques and things like that. You sometimes used to see certain lifting techniques that you would never train to do, but they were quick and easy and you could do them, but they're damaging for both you [care assistant] and the older person. But it's quick and it gets it done … like lifting people into their armpit really, really bad technique (Research fellow).

It's lack of experience, more than skills. They're [care assistants] given the skills, but they don't have the experience… You get this training but you can't see how it's relevant for your job until you're actually doing it and then you need to experience these incidents to be able to do a reflection and see what's the best way to deal with the situation (Senior care assistant).

I had to deal with an awful lot of people that were struggling with the symptoms of dementia, but nobody trained me in that… Where are the priorities when you are training people to work in settings? Is it about being able to provide compassionate care, or is it being able to function in a building so that if it sets on fire you can evacuate the building? (Research fellow).

I'm sure that's the same for you [referring to the researcher] and for me it's about ticking boxes for insurance purposes. So kind of the mandatory training you do for your NHS Trust for your university, I mean that is largely because of insurance purposes. So it's kind of ticking a box (Old age psychiatrist).

I sometimes think as well this when we're trying to find solutions and when I read, you know, different types of books or trainings related to how we [residential care staff] should support social care. Obviously, we're going for your health certificates, your quality certificate, diplomas, all of those information's giving you ideal scenario in ideal world, how you support someone but actually I would love to see one day the book about challenges (Registered manager).

If you had a higher staffing level that would allow staff to actually engage with more training (Old age psychiatrist).

We have a lot of training courses like me I have more than 15 training courses (Health care assistant - dementia department).

Lack of training is the major barrier. I have been within the care home setting with dementia for five years and I have to say that I had incredible training with the company that I first started with but not all companies are very good with their training for dementia. The company I'm with now is an example of that. Their training is not up to par, it's not engaging, it's not informative enough (Senior care assistant).

Not everybody that is in the class are dedicated to listen to the lecturer. Some are there, they are dedicated. They won't know what that lecture is saying so that they can be able to learn some are there but they don't care, they don't. They are not dedicated, they don't practise. That's probably you are in class now after class you have to go home and review what the lecturer said and you

can be able to get more knowledge and make more research and find out the other proper things that is needed so people are different (Health care assistant - dementia department).

I think it's about the budget (Senior care assistant).

Subtheme: Strategies to upskill residential care staff

We created an additional role which is called care delivery coordinator and that person is providing induction. So there's a continuity of the knowledge that is passed onto the staff because we want the same standards. We want the same understanding of the values as well (Registered manager).

Then you probably will sustain it and also use it so that the training just needs to be made fun for them (Old age psychiatrist).

I also think it's about the trainers training, if that makes sense. It goes even higher than just one person training the staff. It's about how that person was trained to deliver the training (Senior care assistant).

And there could also be a value in role modelling a little bit more and that's kind of that's what one of the clinics XXX here has taken up. They've kind of funded a support worker who goes into care homes and role models how to work with people with dementia and we hope that helps a little bit (Old age psychiatrist).

For us induction, because I remember when I was starting my journey with social care, you were given in the past two days for your induction and off you go, you work on the floor where you feel as well, that you don't know your residence (Registered manager).

THEME: LIMITED RESOURCES FROM THE BROADER HEALTHCARE SYSTEM

The care home is not the problem, the problem is the healthcare system in the country that delays those things. Care home is effective because there are regulations (Health care assistant - dementia department).

They [GPs] do not have the capability to attend very fast, or sometimes it may take few weeks, a month, two months until they respond. Obviously when somebody has a severe mental health crisis, then we pressure the doctor as well to follow up with them and we follow up with phone calls and then they realise that this is urgent, so they start prioritising (Registered nurse).

How can somebody [resident] be expelling blood and you tell me that his appointment is next two months? (Health care assistant - dementia department).

So continuity of staff is one of the key things because somebody else wouldn't have picked that up, I don't think (Research fellow).

Making it easy for care homes to access advice from a GP (Geriatrician).

You have nations to set out standards and those standards should be standards of care and there should be a way of evaluating them (Emeritus professor of psychology, and consultant clinical neuropsychologists).

Clearer pathways of care for medical problems that people [residents] have so knowing when to admit them to hospital and when it would be better to keep them in the care home (Geriatrician).

We don't want to have general overview of something just, you know, create specialistic care homes which will you know, support with mental health disorders/dementia. But at the moment

we have a mix of conditions and then, you know, we're not going to be, you know, talking about funding and under funding. But I think for many years as many people will say, social care was left out, isn't it? And we have the effect of it now (Registered manager).

Supplementary Text 1:

Interview Script

Thank you for your continued participation in the study “managing comorbidities in people with dementia in residential care”. Based on your answers and those of the remaining participants in the previous two rounds, we found that the three comorbid conditions most frequently judged as most difficult to manage are: (1) mental health illnesses; (2) delirium; (3) sensory impairment. I will ask you some questions for each of these conditions and, if you only have 20 minutes for the interview, I would ask you to take maximum 3 minutes per issue. If you have more time, please take all the time you need to answer.

Starting from mental illnesses, what makes managing illnesses difficult, and what could be done changed to make mental illnesses more easily manageable?

Moving to delirium, what makes managing delirium difficult, and what could be done/changed to make delirium more easily manageable?

Moving to sensory impairment, what makes managing sensory impairment difficult, and what could be done/changed to make sensory impairments more easily manageable?

Based on your answers and those of all the other participants in the two previous Delphi rounds, we found that the most commonly mentioned issue when dealing with comorbidity in dementia in care/nursing homes is management of symptoms (mentioned by 6 participants). Other common issues mentioned by 5 participants each are medication management, limited staff, and lack of skills among staff.

I will ask you the same questions for each of these issues.

Starting from management of symptoms, what makes symptoms management difficult and how could symptoms be best managed?

Moving to medication management, how could medications be best managed?

Moving to limited staff, what is in your opinion the cause of this issue, and is there a way of reducing it?

Finally, moving to lack of skills among staff, again, what is the cause of this issues, and how could skills be improved among staff?
